# Spatio-Temporal Agnostic Sampling for Imbalanced Multivariate Seasonal Time Series Data: A Study on Forest Fires †

**DOI:** 10.3390/s25030792

**Published:** 2025-01-28

**Authors:** Abdul Mutakabbir, Chung-Horng Lung, Kshirasagar Naik, Marzia Zaman, Samuel A. Ajila, Thambirajah Ravichandran, Richard Purcell, Srinivas Sampalli

**Affiliations:** 1 Department of Data Science, Analytics, and Artificial Intelligence, Carleton University, Ottawa, ON K1S 5B6, Canada; 2 Department of Systems and Computer Engineering, Carleton University, Ottawa, ON K1S 5B6, Canada; chlung@sce.carleton.ca (C.-H.L.); ajila@sce.carleton.ca (S.A.A.); 3 Department of Electrical and Computer Engineering, University of Waterloo, Waterloo, ON N2L 3G1, Canada; snaik@uwaterloo.ca; 4 Research and Development, Cistel Technology, Nepean, ON K2E 7V7, Canada; marzia@cistel.com; 5 Research and Development, Hegyi Geomatics Inc., Nepean, ON K2E 7K3, Canada; rravichandran@hegyigeomatics.com; 6 Faculty of Computer Science, Dalhousie University, Halifax, NS B3H 4R2, Canada; richard.purcell@dal.ca (R.P.); srini@cs.dal.ca (S.S.)

**Keywords:** sensors, under-sampling, deep learning, big data analytics, natural fire disasters, climate change, evolving data, real-time data sampling, nearmiss, SMOTE, multivariate time series

## Abstract

Natural disasters are mostly seasonal and caused by anthropological, climatic, and geological factors that impact human life, economy, ecology, and natural resources. This paper focuses on increasingly widespread forest fires, causing greater destruction in recent years. Data obtained from sensors for predicting forest fires and assessing fire severity, i.e., area burned, are multivariate, seasonal, and highly imbalanced with a ratio of 100,000+ non-fire events to 1 fire event. This paper presents Spatio-Temporal Agnostic Sampling (STAS) to overcome the challenge of highly imbalanced data. This paper first presents a mathematical understanding of fire and non-fire events and then a thorough complexity analysis of the proposed STAS framework and two existing methods, NearMiss and SMOTE. Further, to investigate the applicability of STAS, binary classification models (to determine the probability of forest fire) and regression models (to assess the severity of forest fire) were built on the data generated from STAS. A total of 432 experiments were conducted to validate the robustness of the STAS parameters. Additional experiments with a temporal data split were conducted to further validate the results. The results show that 180 of the 216 binary classification models had an F1score>0.9 and 150 of the 216 regression models had an $R^{2}\;score>0.75$. These results indicate the applicability of STAS for fire prediction with highly imbalanced multivariate seasonal time series data.

## 1. Introduction

The occurrence of natural phenomena, such as forest fires, tsunamis, earthquakes, and cyclones, may lead to natural disasters spanning large areas in different geographical regions. This research presents an under-sampling framework to predict natural disasters by using forest fires as an example. Predictions on the occurrence of these phenomena can be performed using time series, multivariate, multi-source, and seasonal data collected from sensors and satellites [1,2,3,4,5,6,7,8,9,10,11,12,13,14,15]. The data are also recorded over consistent periods, such as hourly, daily, or monthly. Additionally, sensor data are recorded from multiple spatial locations at the same time. When using such data, the challenge of sampling the majority class (non-event data) arises due to the imbalance in data among classes. This is because the event data are spread far across geographic distances and in time, leading to very high non-event data. It is to be noted that event and non-event are used in a general context here, and when used in the context of forest fires, they are referred to as fire and non-fire events (elaborated in Section 4.1).

Natural phenomena, such as forest fires, are essential for ecological integrity. Coexistence with forest fires by allowing prescribed burns can help maintain ecological integrity [16]. However, forest fires may also be caused by humans intentionally or by accident. Further, they can occur naturally by lightning, provided that suitable meteorological conditions exist for ignition [15,17,18]. This paper confines itself to naturally occurring forest fires.

Canadian Geographic pointed out that in the 2023 fire season, as of August 2023, forest fires in Canada had burned 15.2 million hectares compared with 7.1 million hectares burned in 1995 [19]. NASA reported that in early August 2023, more than 300 megatons of carbon emissions were generated due to forest fires in Canada, more than three times that has been generated in recent decades [20]. Hence, it is important to reliably predict forest fires and fire severity.

The data available for forest fire prediction, such as the weather station data, are highly imbalanced with a ratio of 100,000+ non-fire events to 1 fire event. The imbalanced datasets affect forest fire research and prediction. The commonly used techniques, such as NearMiss and Synthetic Minority Over-sampling TEchnique (SMOTE) are explained in detail in Section 2.2. The primary limitation of these techniques is that they are computationally expensive, especially for real-time evolving data. To overcome the challenge of sampling highly imbalanced multivariate seasonal time series data in real time, Spatio-Temporal Agnostic Sampling (STAS) was introduced in [8]. The contributions of this paper are as follows:

1. A mathematical description of multivariate time series event and non-event data is provided along with a description of the STAS framework;
2. The computation speed gained by STAS over two other common sampling algorithms (NearMiss and SMOTE) for real-time applications in natural forest fire disasters is presented through a time complexity analysis;
3. Algorithms for K-Nearest Sensor Data Aggregation and Spatio-Temporal Agnostic Sampling in the STAS framework have been modified for better understandability and readability;
4. Validation of the robustness of the parameters proposed in the STAS framework [8] was conducted through an extensive set of possible parameter values;
5. An additional set of experiments based on a temporal split of fire and non-fire event data were conducted demonstrating that the binary classification and regression models used in STAS are not impacted by current or future events during training.

The available tools for forest fire prediction may not be reliable due to the possibility of regional bias. Univariate data are not suitable for predicting forest fires and severity. Lightning, wind, groundwater level, precipitation, elevation, and slope influence forest fires and their severity [21]. In this research, a total of 432 experiments were conducted with 216 experiments each for binary classification and regression to demonstrate that STAS can be used with highly imbalanced multivariate seasonal time series data to identify the change in features with high prediction accuracy.

The rest of this paper is organized as follows: Section 2 presents the related work and background. Section 3 discusses the datasets and the methodology is presented in Section 4. The experiments along with the results are presented in Section 5. A discussion of STAS and concluding remarks are provided in Section 6 and Section 7, respectively.

## 2. Literature Review and Background

This section presents some of the available literature on the main topics this research tries to cover. The available literature is categorized into themes, such as sampling techniques, fire weather index, and forest fire prediction models, which are further subdivided. This section also presents the terminology used and the background of the study.

### 2.1. Terminology

The following are some of the terms used in this study:

- **Time Series**: Data points recorded over a period of time, in successive order with regular time intervals.
- **Multivariate**: A dataset with more than one independent variable for any given data point.
- **Seasonality**: Time series data points having regular and periodic changes that occur at near-constant time intervals.

### 2.2. Sampling Techniques

In imbalanced data, the class with a higher number of samples is referred to as the majority class, and the class with a lower number of samples is referred to as the minority class. In natural phenomena, such as forest fires, the majority of the data points are for non-fire data. When models are trained on such imbalanced data, they tend to accurately predict only non-fire events [12,13].

Sampling is a technique used to select a subset from a population. Sampling has been frequently used for sensor data [11,12,13,22,23,24,25]. The common approaches are to either under-sample the majority class or over-sample the minority class, or a combination of both. Some sampling techniques are random sampling, NearMiss, SMOTE, and Information Based Optimal Subdata Selection (IBOSS) [23,24,25].

#### 2.2.1. Random Sampling

Random sampling is one of the most popular under-sampling techniques, and it was used in [13], where a random sample of data points was taken from the majority class. It was found that models trained using randomly sampled data could not identify fire events but were able to correctly classify non-fire events [13].

#### 2.2.2. NearMiss

The NearMiss approach is another popular technique used in under-sampling [26,27,28]. It was used in previous research [12,13] to undersample the data. There are three types of NearMiss approaches, as explained in the following passage. NearMiss-1 selects data points from the majority class with the smallest average distance to the three closest data points from the minority class. NearMiss-2 selects data points from the majority class that has the smallest average distance to the three furthest data points from the minority class. In NearMiss-3, for every minority class data point, a given number of majority class data points are selected that are closest to the minority class data point. The number of majority class data points can be selected while running the algorithm. In [12], it was seen that with under-sampling, as the ratio of fire and non-fire events approached 1:1, the models started to identify fire events but did not accurately identify non-fire events as compared with no sampling. An effective under-sampling ratio was hard to determine.

#### 2.2.3. Synthetic Minority Over-Sampling TEchnique

SMOTE is a statistical technique used to address the issue of class imbalance by over-sampling the minority class. It was used in [12]. It first selects a sample *a* from the minority class. Then, it selects *x* minority class samples closest to *a*. Finally, it uses linear interpolation between *a* and *x* to generate more samples. Over-sampling produced better results when compared with under-sampling [12].

#### 2.2.4. Spatio-Temporal Agnostic Sampling

A common problem with machine learning models trained on extremely imbalanced data is that they favor predicting the majority class. In [12], when the models were trained without sampling the data, the models mostly predicted the non-fire. STAS [8], instead of letting the models identify between majority and minority class, transforms the problem to ***identify the change in features over a time frame M for an event to occur***.  It is a repeatable under-sampling technique that generates a balanced dataset for large-scale evolving data and ensures seasonality does not impact prediction and can be applied to sensor and satellite data. We proposed and validated the STAS framework to predict forest fires using weather data in [8,9] and later extended it to federated learning in [10]. In [9], it was also validated that features completely independent of forest fires, i.e., hydrometric data do not increase or decrease the performance of models. In [8], the parameters for STAS used in the framework were N=7,14,30; M=3,5,6,7,9,12; and K=1,3,5, where N represents the number of past days’ information each feature has in a data point, M represents how many months back to select a non-fire event, and K determines how many stations’ values are averaged to make predictions for sensor data.

### 2.3. Fire Weather Index

The Canadian Fire Weather Index (FWI) system plays a major role in forest fire prediction. It is a part of the Canadian Wildland Fire Information System (CWFIS), which provides the fire danger level values for the entire country. It is the principal source of fire intelligence for forest fire management services [21]. It has been successfully migrated to other parts of the world, such as Poland [29], Portugal [30], China, Italy [31], Indonesia, Spain, and the United States. The Canadian FWI system has been extensively applied in other research [21,29,31,32,33]. Stocks et al. [32] provided a good background to this research. It was highlighted in [33] that wind, temperature, humidity, and rain are useful in predicting fire spread.

**Figure 1:**
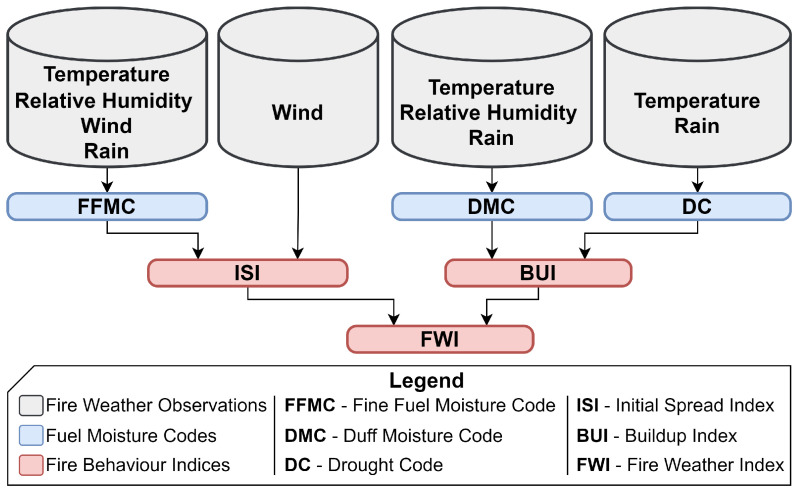
Structure of Canadian FWI system adapted from [34].

The Canadian FWI system includes fire weather behavior based on temperature, relative humidity, wind, and rain. A pictorial representation of the FWI system is shown in Figure 1. The Canadian FWI system has six components divided equally into fuel moisture codes and fire behavior indices. The fuel moisture codes are Fine Fuel Moisture Code (FFMC), Duff Moisture Code (DMC), and Drought Code (DC) shown in blue in Figure 1. The three fire behavior indices are Initial Spread Index (ISI), BuildUp Index (BUI), and FWI, shown in red in Figure 1.

In Canadian FWI, fuel moisture codes are calculated using similar features. For example, all three (FFMC, DMC, and DC) use temperature and rain to calculate their values. FFMC and DMC have relative humidity in common as well. Later, the fire behavior indices are calculated using FFMC, DMC, and DC. This leads to redundant calculations of readings, such as temperature, relative humidity, and rain. Further, the equations used to calculate these values have seasonal constants that need to be calibrated with intervention from human experts in the field. A detailed overview of the calculations can be found in [34]. Additionally, FWI does not consider that region’s forestry data. This may predict fire danger even if no forests exist in the region. Meanwhile, in [10], the STAS models proved effective in diverse environments. Features similar to the Canadian FWI system were used in STAS; however, human calibration was not needed.

### 2.4. Forest Fire Prediction Models

As concluded in [35], the National Fire Data Base (NFDB) points and polygons give the best estimates of fire season start and end dates. The aim of [35] is to examine the trends in fire-regime changes. Research on neural network forecasting for seasonal and trend time series [36] showed that neural networks do not capture seasonal or trend variations with raw data. The model proposed in [37] was developed to predict the severity of small and frequent fires using meteorological input. The researchers proposed using a Support Vector Machine (SVM) and Random Forest (RF). Much earlier, [38] presented a forest fire risk prediction algorithm based on SVM with only meteorological data.

The research in [11,37,38,39,40,41,42,43] focuses on using meteorological data to make predictions. These studies are limited to a particular region. The research in [44,45,46] considers lightning as the primary factor for predicting natural forest fires. These too are limited to narrow regions. This leads to regional bias in forest fire predictions. Hence, it is proposed to use large geographic data in this research.

### 2.5. Metrics

There are different types of performance metrics for classification tasks with machine learning, such as accuracy, recall (sensitivity), specificity, and precision. Accuracy is a common metric used in classification.

Recall indicates what proportion of the data belonging to a class is classified correctly in that class by the classifier. The formula for recall is given in Equation (1).

(1) $$Recall=\frac{True\;Positive}{True\;Positive+False\;Negative}$$

Precision tells us how many true positives were there among all the true positives and false positives. The formula for precision is shown in Equation (2).

(2) $$Precision=\frac{True\;Positive}{True\;Positive+False\;Positive}$$

F1score (F1) is a standard machine learning metric used in classification models. It is the harmonic mean of precision and recall. The value of F1 is considered excellent if it is in the range (0.9−1], good if in the range (0.8−0.9], poor if in the range (0.5−0.8], and bad if in the range [0−0.5]. The formula for F1 is shown in Equation (3).

(3) $$F1=2\times \frac{Precision\times Recall}{Precision+Recall}$$

$R^{2}\;score$ ($R^{2}$) is a metric used in evaluating the performance of a regression-based machine learning model by measuring the variation in the dependent variables on the independent variables. It measures the goodness of fit. Significant variance is explained if the $R^{2}$ is in the range (0.75−1], good variance is explained if in the range (0.5−0.75], and little or no variance is explained if in the range [0−0.5]. $R^{2}$ can be calculated using the Equation (4) where *n* is the number of samples, $y_{i}$ is the $i^{th}$ target value, $\overset{´}{y_{i}}$ is the $i^{th}$ predicted value, and $\mu_{y}$ is the mean of target values.

(4) $$R^{2}=1-\frac{\sum_{i=1}^{n}{\left(y_{i}-\overset{´}{y_{i}}\right)}^{2}}{\sum_{i=1}^{n}{\left(y_{i}-\mu_{y}\right)}^{2}}$$

This paper proposes to use F1 as a metric for classification tasks because F1 provides an understanding of the model’s performance with imbalanced data and incorporates precision and recall. Also, $R^{2}$ is proposed as a metric for regression models. This is because $R^{2}$ measures the goodness of fit for regression models, given a set of inputs.

## 3. Datasets

This section describes the datasets used in this research. Two sources of data were used, the Canadian National Fire Database (CNFDB) and the Canadian Weather Energy and Engineering Datasets (CWEEDS), which are summarized in the following subsections. The final dataset used to predict forest fires is constructed by merging the CWEEDS and CNFDB datasets (Table 1), as explained in Section 4.2. In Table 2 we can see the CWEEDS metadata features used in the preprocessing. The size of input for modeling will vary based on the parameter N in STAS. Each data point in the final dataset will have N historic days of information for all the features listed in Table 3. The final dataset will have a 1:1 ratio for fire to non-fire events. The final dataset covers the entire country of Canada from 1998 to 2018, with a granularity of a day. The final dataset contains roughly **30,000 data points** approximately two times the number of naturally occurring forest fires (roughly 15000) in the CNFDB.

### 3.1. Canadian National Fire Database

**Figure 2:**
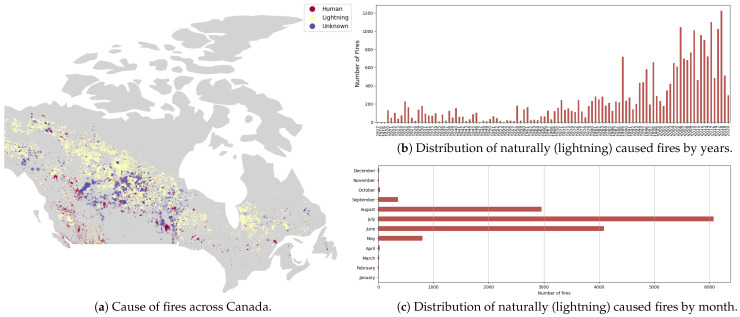
Distribution of fires across Canada in Canadian National Fire Database (CNFDB).

The CNFDB dataset [47] provides historical information on fire events for the entire Canada from 1917 to 2020. The database has been updated over time. Since this dataset is a ledger of forest fires across Canada, it does not have temporal granularity. Instead, it has a spatial extent of complete Canada. It also includes information on the shape, time, cause, and location of the fire events. The dataset is available as a shapefile. A visual representation of the distribution of the fires in the CNFDB is given in Figure 2. The causes of fires across Canada is shown in Figure 2a. The fires burned by human factors are shown in red, the naturally (lightning) caused fires are shown in yellow, and the ones for which the cause was unknown are shown in purple. In Figure 2b, we see the distribution of naturally caused fires over the years in CNFDB. In Figure 2c, we see the distribution of naturally caused fires by months. Most fires occur between June and August and are seasonal.

There are many features available in CNFDB. When making predictions using this dataset, some features, such as MORE_INFO, POLY_DATE, and ACQ_DATE, are not needed. Apart from this, there are redundant features in the dataset, such as YEAR, MONTH, and DAY, which are represented in REP_DATE. Table 1 lists the features from the CNFDB dataset used in this research.

### 3.2. Canadian Weather Energy and Engineering Datasets

The CWEEDS dataset [48] available from 1998 to 2018 has an hourly granularity. A document detailing the data can be found in [48]. CWEEDS was chosen as an ideal dataset as it provides features similar to the ones used in Canadian FWI. This dataset provides a historical record of the meteorological (weather) data of Canada for multiple weather stations. It contains one file for all the weather station metadata, and then each weather station’s data are contained in a separate file. The CWEEDS weather station metadata fields used in this research are presented in Table 2.

The preprocessed weather station data features are provided in Table 3 along with their units. Column three specifies the preprocessing done, while column four specifies the final preprocessed unit. Further, in the unprocessed weather station data files, each of the features has a flag value associated with it. The flag provides information on how the recording was made. This information was discarded in the processed dataset. The features having digit codes as their unit, such as sky layers and weather, are a combination of multiple-digit values (shown as dark purple Table 3) and are expanded in the final preprocessed dataset.

## 4. Methodology

This section first describes event and non-event data and provides a table for the notation used in this research. Then, the STAS framework is discussed in detail. Following this, a time complexity analysis of the STAS framework and comparison with NearMiss and SMOTE is presented. Finally, the deep learning model architecture used is discussed.

### 4.1. Fire and Non-Fire Events

In this research, event(s) and non-event(s) are referred to in the context of any natural disaster. When used in the context of forest fires, they are referred to as fire and non-fire event(s). Consider a single forest fire event *f* that belongs to a set *F* of all forest fire events. Here, $f_{s}$, $f_{g}$, and $f_{l}$ represent REP_DATE (start date of *f*), GEOMETRY (final 2 dimensional burnt area of *f*), and center of GEOMETRY of *f*, respectively, from Table 1.

$$F=\{f|f\;\mathrm{is}\;\mathrm{forest}\;\mathrm{fire}\;\mathrm{event}\;\mathrm{starting}\;\mathrm{at}\;f_{s}\;\mathrm{with}\;a\;\mathrm{final}\;\mathrm{burned}\;\mathrm{area}\;f_{g}\;\mathrm{having}\;\mathrm{center}\;\mathrm{at}\;\mathrm{location}\;\mathrm{point}\;f_{l}\}$$

Let *S* be the set of sensors (weather stations in case of forest fires) as shown in Table 2.

$$S=\{s|s\;\mathrm{contains}\;\mathrm{metadata}\;\mathrm{of}\;\mathrm{the}\;\mathrm{sensor}\;\mathrm{located}\;\mathrm{at}\;s_{l}\}$$

Finally, consider *W* a set of multivariate time series sensor data as shown in Equation (5). The sensors record *p* features. In this research, p=31, since weather station data (Table 3) is used. Each sensor data *w* in the set *W* will record time series data for *p* features between the time frame t=0 until $t=t_{max}$. Here, t=0 indicates the oldest time recorded among all the sensors in *S*, while $t_{max}$ is the latest time recorded among all the sensors in *S*. For forest fires *t* increments in days. The value of *t* belongs to the set of natural numbers (N). If there is no reading at a certain value of *t*, then the missing values are padded with 0 to keep a consistent input feature size.

(5) $$\begin{array}{cc} W=\{w & \in R^{p\times (t_{\max}+1)}|w\;\mathrm{is}\;\mathrm{multivariate}\;\mathrm{time}\;\mathrm{series}\;\mathrm{data}\}\\ w & =\left[\begin{array}{cccc} w_{1,t=0} & w_{1,t=1} & \cdots & w_{1,t=t_{max}}\\ w_{2,t=0} & w_{2,t=1} & \cdots & w_{2,t=t_{max}}\\ \vdots & \vdots & \ddots & \vdots \\ w_{p,t=0} & w_{p,t=1} & \cdots & w_{p,t=t_{max}} \end{array}\right] \end{array}$$

Let g(·) be a mapping from set *S* to *W* such that it returns sensor data *w* for a given sensor *s* as shown in Equation (6).

(6) g(·):S→W

K is the number of nearest sensors to consider for a single forest fire *f*. Therefore, K belongs to N (K∈N) and is significantly smaller than the size of the set *S* (K≪|S|). Then, a set of K sensors $S_{k}$ for a given forest fire *f* can be defined as seen in Equation (7), where $∥s_{l}-f_{l}∥$ is the Euclidean distance between the location of sensor *s* and the center of $f_{g}$ of a forest fire *f*. It is also necessary that the sensors(s) selected should be operational at time $f_{s}$.

(7) $$S_{k}=\{S_{k}\subset S,|S_{k}|=K,\arg\min_{s\in S}∥s_{l}-f_{l}∥\}$$

Therefore, the list of multivariate time series sensor data $W_{k}$ acquired for each sensor in $S_{k}$ can be defined as seen in Equation (8). ${\bar{w}}_{k}$ is the average of the values in $W_{k}$ as seen in Equation (9), where $w_{i}$ is $i^{th}$ sensor data matrix in $W_{k}$.

(8) $$W_{k}=\left[g\left(s\right)|s\in S_{k}\right]$$

(9) $${\bar{w}}_{k}=\frac{1}{K}\sum_{i=1}^{K}w_{i}$$

In STAS, N specifies the number of past days incorporated in a single event or non-event. It is measured in discrete units; hence, N∈N. In the case of forest fires, it is measured in days. M is the time difference for a non-event to be considered, similarly M∈N. For forest fires, it is considered in months.

In this research, a single **event** (data point) $\hat{e}$ is defined as a multivariate time series data extracted from ${\bar{w}}_{k}$, starting at the occurrence of a natural disaster and going N historical records in the past in reverse order, i.e., a **fire event **$\hat{e}\in R^{p\times (N+1)}$ is the time series extracted from ${\bar{w}}_{k}$ for a forest fire *f* between the time frame $\left[f_{s},f_{s}-N\right]$, as shown in Equation (10). There are N+1 days, since $f_{s}$ is included as well.

(10) $$\begin{array}{cccc} \; & \; & \hat{e} & =\left[\begin{array}{cccc} {\bar{w}}_{1,t=f_{s}} & {\bar{w}}_{1,t=f_{s}-1} & \cdots & {\bar{w}}_{1,t=f_{s}-N}\\ {\bar{w}}_{2,t=f_{s}} & {\bar{w}}_{2,t=f_{s}-1} & \cdots & {\bar{w}}_{2,t=f_{s}-N}\\ \vdots & \vdots & \ddots & \vdots \\ {\bar{w}}_{p,t=f_{s}} & {\bar{w}}_{p,t=f_{s}-1} & \cdots & {\bar{w}}_{p,t=f_{s}-N} \end{array}\right] \end{array}$$

Similarly, a single **non-event** (data point) $\overset{ˇ}{e}$ is defined as a multivariate time series data extracted from ${\bar{w}}_{k}$, starting M months prior to the occurrence of a natural disaster and going until N historical records in the past in reverse order, i.e., a **non-fire event **$\overset{ˇ}{e}\in R^{p\times (N+1)}$ is a time series extracted from ${\bar{w}}_{k}$ for a forest fire *f* between the time frame $\left[f_{s}-M,f_{s}-M-N\right]$, as seen in Equation (11).

(11) $$\overset{ˇ}{e}=\left[\begin{array}{cccc} {\bar{w}}_{1,t=f_{s}-M} & {\bar{w}}_{1,t=f_{s}-M-1} & \cdots & {\bar{w}}_{1,t=f_{s}-M-N}\\ {\bar{w}}_{2,t=f_{s}-M} & {\bar{w}}_{2,t=f_{s}-M-1} & \cdots & {\bar{w}}_{2,t=f_{s}-M-N}\\ \vdots & \vdots & \ddots & \vdots \\ {\bar{w}}_{p,t=f_{s}-M} & {\bar{w}}_{p,t=f_{s}-M-1} & \cdots & {\bar{w}}_{p,t=f_{s}-M-N} \end{array}\right]$$

This makes the data agnostic to spatial resolution. The dataset thus produced, for a single combination of values of K, N, and M, will have a 1:1 ratio of fire and non-fire events, which is equal to the number of forest fires in *F*. The notation used is described in Table 4.

### 4.2. Framework

**Figure 3:**
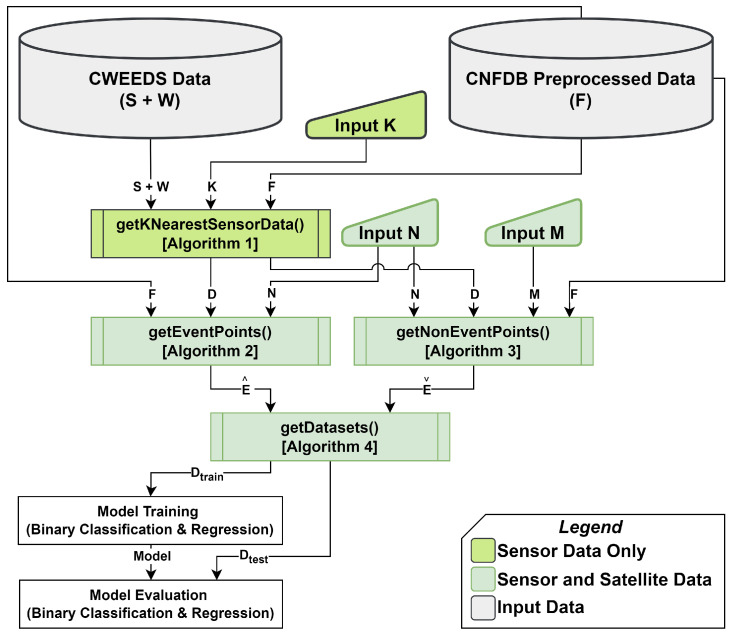
STAS framework adapted from [8].

The framework for forest fire prediction using STAS is shown in Figure 3. A detailed description of the framework is presented in [8]. Table 4 describes the variables used in Algorithms 1–4. Algorithm 1, and K are only needed for sensor data processing. The rest are needed for both sensor and satellite data. This is shown with different shades of green in Figure 3. The values of K, *S*, *W*, and *F* are provided as input to Algorithm 1. In Algorithm 1, K-nearest weather stations from the set *S* are selected for every fire *f* in *F*. The output *D* contains the average of K multivariate time series weather data for each forest fire.

The value of N is selected and is provided as input to Algorithm 2 along with *D* from Algorithm 1 and the set *F*. Algorithm 2 extracts fire events by limiting each value in *D* to the time frame $\left[f_{s},f_{s}-N\right]$ by looping over all values in *D* and *F* as a combined set (similar to the zip function in Python). For Algorithm 3, the value of M is selected and provided as input along with N, *F*, and *D* from Algorithm 1. Algorithm 3 extracts non-fire events by limiting each value in *D* to the time frame $\left[f_{s}-M,f_{s}-M-N\right]$ by looping over all values in *D* and *F* as a combined set. For example, consider M=3, N=30 for a forest fire event *f* starting at $f_{s}=$ 14 August 2023. Then, multivariate time series for the fire event $\hat{e}$ is extracted between 14 August 2023 $\left(t_{start}\right)$ and going backward until 15 July $2023\;(t_{end})$. The multivariate time series data for the non-fire event $\overset{ˇ}{e}$ is extracted between 14 May 2023 $\left(t_{start}\right)$ going backward until 14 April 2023$\left(t_{end}\right)$.
**Algorithm 1 **K-Nearest Sensor Data AggregationAll the symbols and notations are described in Table 4.     **Input**: K, *S*, *W*, *F*     **Output**: *D*  1:**procedure** getKNearestSensorData(K, *S*, *W*, *F*)  2:    D←∅  3:    **for** f∈F **do**  4:        $f_{l}\leftarrow$ center location of $f_{g}$ in *f*  5:        $S_{k}\leftarrow K$-nearest sensor to $f_{l}$               ▹ Using Equation (7)  6:        $W_{k}\leftarrow \emptyset$  7:        **for** $s\in S_{k}$ **do**  8:           w←g(s)                                    ▹ As seen in Equation (6)  9:           $W_{k}\leftarrow$ add *w*10:       **end for**11:        ${\bar{w}}_{k}\leftarrow$ average $W_{k}$                              ▹ Using Equation (9)12:        D← add ${\bar{w}}_{k}$ to *D*13:    **end for**14:**end procedure**

**Algorithm 2** Event Points Extraction
All the symbols and notations are described in Table 4.
     **Input**: N, *F*, *D*
     **Output**: $\hat{E}$
  1:**procedure**  getEventPoints(N, *F*, *D*)  2:    $\hat{E}\leftarrow \emptyset$  3:    **for** $\left({\bar{w}}_{k},f\right)\in \left(D,F\right)$ **do**                         ▹ Zip of sets (*D*, *F*)  4:        $f_{s}\leftarrow$ start date of natural fire disaster *f*  5:        $t_{start}\leftarrow f_{s}$  6:        $t_{end}\leftarrow f_{s}-N$  7:        $\hat{e}\leftarrow {\bar{w}}_{k}$ between $\left[t_{start},t_{end}\right)$  8:        $\hat{E}\leftarrow$ add $\hat{e}$ to $\hat{E}$  9:    **end for**10:
**end procedure**



The outputs from Algorithm 2 ($\hat{E}$) and Algorithm 3 ($\overset{ˇ}{E}$) are provided as inputs to Algorithm 4. The target values are appended based on the prediction being made. In the case of binary classification, $\hat{E}$ gets 1 and $\overset{ˇ}{E}$ gets 0, while in the case of regression, $\hat{E}$ gets the area burnt (severity) by forest fires and $\overset{ˇ}{E}$ gets 0. Any spatial or temporal information, such as date, time, latitude, and longitude, is removed to make the data spatio-temporal agnostic. The data are standardized using mean and standard deviation. Finally, the data are randomly sampled and split into $D_{train}$ and $D_{test}$, which are used for training and testing of deep learning models, respectively. $D_{train}$ gets 80% of the fire and non-fire events, while $D_{test}$ gets 20%. $D_{train}$ is then used to train models described in Section 4.4. Once the model is trained, $D_{test}$ is used to evaluate the model.
**Algorithm 3** Non-Event Point ExtractionAll the symbols and notations are described in Table 4.     **Input**: N, M, *F*, *D*     **Output**: $\overset{ˇ}{E}$  1:**procedure**  getNonEventPoints(N, M, *F*, *D*)  2:    $\overset{ˇ}{E}\leftarrow \emptyset$  3:    **for** $\left({\bar{w}}_{k},f\right)\in \left(D,F\right)$ **do**                         ▹ Zip of sets (*D*, *F*)  4:        $f_{s}\leftarrow$ start date of natural fire disaster *f*  5:        $t_{start}\leftarrow f_{s}-M$  6:        $t_{end}\leftarrow f_{s}-M-N$  7:        **if** $t_{s}\neq f_{s}\forall f\in F$ **then**  8:           $\overset{ˇ}{e}\leftarrow {\bar{w}}_{k}$ between $\left[t_{start},t_{end}\right)$  9:           $\overset{ˇ}{E}\leftarrow$ add $\overset{ˇ}{e}$ to $\overset{ˇ}{E}$10:        **end if**11:    **end for**12:**end procedure**

**Algorithm 4** Spatio-Temporal Agnostic Sampling
All the symbols and notations are described in Table 4.
     **Input**: $\hat{E}$, $\overset{ˇ}{E}$
     **Output**: $D_{train}$, $D_{test}$
  1:**procedure**  getDatasets($\hat{E}$, $\overset{ˇ}{E}$)  2:    D←∅  3:    $\hat{E}\leftarrow$ add target values to $\hat{E}$  4:    D← add $\hat{E}$ to *D*  5:    $\overset{ˇ}{E}\leftarrow$ add target values to $\overset{ˇ}{E}$  6:    D← add $\overset{ˇ}{E}$ to *D*  7:    D← delete spatio-temporal data from dataset  8:    D← standardize *D*  9:    $D_{train},D_{test}\leftarrow$ partition *D* to get train and test dataset10:
**end procedure**



### 4.3. Time Complexity Analysis

Consider a dataset *A* with points either belonging to the majority class $A_{maj}$ or the minority class $A_{min}$. Each value *a* belonging to *A* is *p* dimensional.

(12) $$A=\{a|a\in R^{p},\;a\in A_{maj}\;\mathrm{or}\;a\in A_{min}\}$$

By definition, the size of $A_{maj}$ is significantly larger than that of $A_{min}$. We can consider the size of a set of natural disasters (forest fires) *F* to be approximately equal to that of the minority class. This can be seen in Equation (13).

(13) $$\begin{array}{c} |A_{min}|\ll |A_{maj}|\\ \left|F|\approx \right|A_{min}|\ll |A_{maj}| \end{array}$$

Majority and minority class data are taken from the values recorded by the sensors in set *S*. Each sensor records for a long time frame. Therefore, the size of *S* is significantly lower than the size of $A_{maj}$, as seen in Equation (14).

(14) $$\left|S|\ll \;\right|A_{maj}|$$

It is hard to compare the sizes of sets *F* and *S*, since they heavily depend on the time frame being considered and may vary based on the natural disaster under study. It is known that the data used to make the predictions are multivariate (p≥2). The size of set *F* may be smaller than the size of set *S*, but we can assume that p×|F| is larger than the size of *S*. If p is significantly larger than 2, then we can assume Equation (15).

(15) $$\begin{array}{cccc} \; & p\geq 2 & \\ p\times |F| & >|S| & \\ \frac{p}{2}\times \left|F\right| & >|S| & \; & \mathrm{If}\;p\gg 2 \end{array}$$

The Big-O (*O*) notation is used for time complexity in the worst case. To compute the time complexity for the distance between two sets of points which are *p*-dimensional, we need to obtain the difference between each dimension for each pair of points in the two sets $A_{1}$ and $A_{2}$. This can be represented by Equation (16). For sorting, the best worst-case time complexity is for Heap Sort, as seen in Equations (17). In Equation (16) and (17), || represents the size of the set.

(16) $$T_{dist}=O(p\times |A_{1}\left|\times \right|A_{2}\left|\right)$$

(17) $$T_{sort}=O(|A_{3}\left|\times \log\right|A_{3}\left|\right)$$

It should be noted that time complexity for distance and sorting calculated in Section 4.3.1–Section 4.3.3 uses Equations (16) and (17). The notation $T_{dist}$ and $T_{sort}$ is used in all subsections, but the values derived are respective to the section.

#### 4.3.1. STAS

For Algorithm 1, we calculate the distance using Equation (7) and then sort computed distances to find the shortest distance. Then, we compute both $T_{dist}$ and $T_{sort}$ for STAS using Equations (16) and (17), respectively. The distance is compared between sets *F* and *S* in two dimensions. Hence, the distance time complexity for Algorithm 1 can be seen in Equation (18).

(18) $$\begin{array}{ccc} T_{dist} & =O(p\times |A_{1}|\times |A_{2}|) & \\ \; & =O(2\times |S|\times |F|) \end{array}$$

For sorting, ordering is applied |F| times to values in *S* to obtain the top K values as seen in Equation (19). Here, $|A_{3}|$ for the sorting complexity in Equation (17) is |S|×|F|.

(19) $$\begin{array}{cc} |A_{3}| & =|S|\times |F|\\ T_{sort} & =O(|A_{3}|\times \log|A_{3}|)\\ \; & =O\left(\left(|S|\times |F|\right)\times \left(\log\left(|S|\times |F|\right)\right)\right) \end{array}$$

Algorithms 2 and 3 loop over the event data points. Therefore, the complexity can be considered linear, as seen in Equation (20). The time complexity of Algorithm 4 can be ignored, as it is of constant time.

(20) $$T_{extr}=O\left(|F|\right)$$

In STAS, if sensor data are considered, then we need to add the time complexity of Equations (18)–(20). Equation (20) is added twice, since we consider it for Algorithms 2 and 3. If satellite data are considered, only Equation (20) can be taken.

(21)O(STASsensor)=Tdist+Tsort+(2×Textr)(22)O(STASsatellite)=2×Textr

#### 4.3.2. NearMiss

A description of the NearMiss algorithm is given in Section 2.2. The distance is compared in p dimensions, and the data are sorted to obtain either the farthest or nearest values. Further, the distance between the majority and minority classes is compared. Hence, the time complexity for distance comparison can be seen in Equation (23).

(23) $$\begin{array}{cccc} T_{dist} & =O(p\times |A_{1}|\times |A_{2}|) & \;\\ \; & =O(p\times |A_{min}|\times |A_{maj}|) & \\ \; & =O(p\times |F|\times |A_{maj}|) & \; & \mathrm{Using}\;\mathrm{Equation}\;(13) \end{array}$$

For sorting, we have to order the distance comparison between the majority and minority classes. Hence, the set $A_{3}$ in Equation (17) is $|A_{min}\left|\times \right|A_{maj}|$. Therefore, the time complexity for sorting is presented in Equation (24),

(24) $$\begin{array}{cc} T_{sort} & =O(|A_{3}|\times \log|A_{3}|)\\ \; & =O\left(\left(|A_{min}\left|\times \right|A_{maj}|\right)\times \left(\log\left(|A_{min}\left|\times \right|A_{maj}|\right)\right)\right)\\ \; & =O\left(\left(\left|F|\times \right|A_{maj}|\right)\times \left(\log\left(\left|F|\times \right|A_{maj}|\right)\right)\right) \end{array}$$

Then, the time complexity of NearMiss will be the sum of Equations (24) and (23), and it is shown in Equation (25).

(25) $$\begin{array}{cc} O(NearMiss) & =T_{dist}+T_{sort} \end{array}$$

#### 4.3.3. SMOTE

SMOTE is described in Section 2.2. First, a distance comparison is made among the data in the minority class. The time complexity for which is shown in Equation (26).

(26) $$\begin{array}{cc} T_{dist} & =O(p\times |A_{1}|\times |A_{2}|)\\ \; & =O(p\times |F|\times |F|)\\ \; & =O(p\times |F{|}^{2}) \end{array}$$

For sorting, we have to order the distance comparison between the minority class data. Hence, the set $A_{3}$ in Equation (17) is $|A_{min}\left|\times \right|A_{min}|$. Therefore, the time complexity for sorting can be seen in Equation (27).

(27) $$\begin{array}{cc} T_{sort} & =O(|A_{3}|\times \log|A_{3}|)\\ \; & =O\left(|A_{min}\left|\times \right|A_{min}|\times \log\left(|A_{min}\left|\times \right|A_{min}|\right)\right)\\ \; & =O\left(|A_{min}{|}^{2}\times \log\left(|A_{min}{|}^{2}\right)\right)\\ \; & =O(2\times |A_{min}{|}^{2}\times \log|A_{min}|)\\ \; & =O(2\times |F{|}^{2}\times \log|F|)\;\;\;\mathrm{Using}\;\mathrm{Equation}\;(13) \end{array}$$

Finally, interpolation is performed. The time complexity for interpolating points can be considered linear. The interpolation is performed for every minority class sample *x* times. *x* is a large value to account for the difference in size between the majority and minority classes. The interpolation time complexity can be seen in Equation (28).

(28) $$\begin{array}{cc} T_{inter\;p} & =O(x\times |A_{min}|)\\ \; & =O(x\times |F|) \end{array}$$

Therefore, the time complexity of SMOTE will be the sum of Equations (26)–(28).

$$\begin{array}{cc} O(SMOTE) & =T_{dist}+T_{sort}+T_{inter\;p} \end{array}$$

#### 4.3.4. Comparison

When comparing the STAS framework with NearMiss, we can say that the time complexity for the STAS framework is lower than NearMiss with either the sensor or satellite data, since $|A_{maj}|$ is used in NearMiss for Equations (23) and (24). From Equations (13) and (14), we know that $|A_{maj}|$ is significantly larger than either |S| or |F|. Therefore,

$$\begin{array}{cccc} O(STAS_{sensor}) & \ll O(NearMiss) & \; & \mathrm{Since}\;\mathrm{Equation}\;\left(14\right)\\ O(STAS_{satellite}) & \ll O(NearMiss) & \; & \mathrm{Since}\;\mathrm{Equation}\;\left(14\right) \end{array}$$

When comparing the STAS framework with SMOTE, we know that for satellite data, the time complexity for STAS is lower than SMOTE, since STAS with satellite data is computed in linear time.

$$\begin{array}{cc} O(STAS_{satellite}) & \ll O(SMOTE) \end{array}$$

If the sensor data are very high-dimensional, then using Equation (15), we can say that the STAS framework is faster than SMOTE. Also, it is to be noted that *x* is a large value in Equation (28). Otherwise, the time complexity is almost similar.

$$\begin{array}{c} O\left(STAS_{sensor}\right)\left\{\begin{array}{cc} <O(SMOTE) & \mathrm{using}\;\mathrm{Equation}\;(15),\;\mathrm{if}\;p\gg 2\\ \approx O(SMOTE) & \mathrm{otherwise} \end{array}\right. \end{array}$$

### 4.4. Modeling

**Figure 4:**
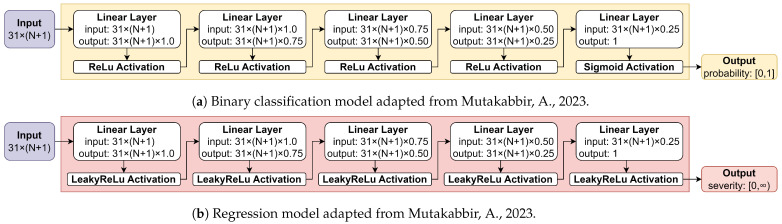
Deep learning models adapted from [8].

In this research, a binary classification model is used to classify data into fire or non-fire events. In our previous research [8], the hypothesis is that the STAS deep learning models will learn the change in features that are likely to cause a fire over the time frame M. The model architecture is depicted in Figure 4a. The binary classification model has an activation of ReLU for all its hidden layers and input layer while having an activation of Sigmoid for the output layer. Target values are set to 1 for fire events ($\hat{e}$), whereas they are set to 0 for non-fire events ($\overset{ˇ}{e}$), as discussed in Section 4.2. Binary Cross-Entropy (BCE) is used as a loss function in this model along with the Adam optimizer. Further details are presented in [8]. Various learning rates ranging from 0.01 to 0.0000001 were tested while developing the models. The learning rate of 0.00001 showed the best results. The models converged fast (<100 epochs). F1 is used to evaluate the models.

Figure 4b illustrates a regression model architecture. It was used to predict the severity of a fire event. The regression model had an activation of LeakyReLU with a negative slope of 0.01 for all its layers. The target value was set to the area burned for fire events ($\hat{e}$) and 0 for non-fire events ($\overset{ˇ}{e}$) as discussed in Section 4.2. Mean Square Error (MSE) was used as a loss function in this model along with the Stochastic Gradient Descent (SGD) optimizer. Further details are presented in [8]. Different learning rates and epochs were tested. It was found that a learning rate of 0.0001 was ideal for all the models. The models converge very slowly (>5000 epochs). $R^{2}$ was used, since it tests for goodness of fit.

## 5. Experiments and Results

**Figure 5:**
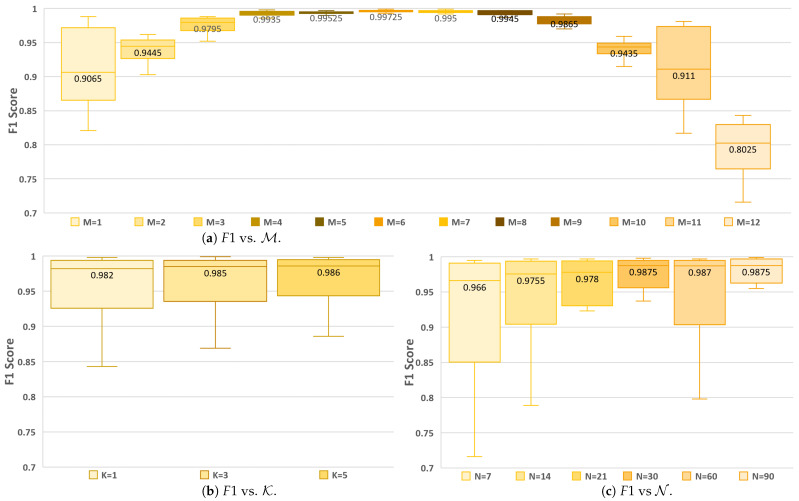
Comparison of the impact of M, K, and N on the binary classification models.

**Figure 6:**
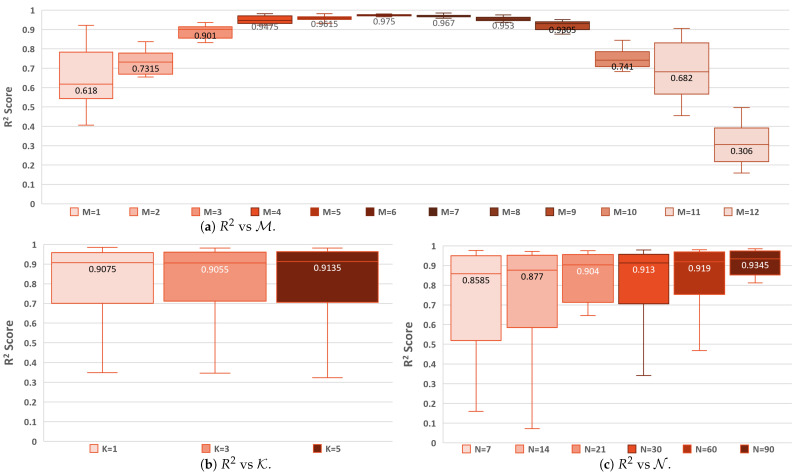
Comparison of the impact of M, K, and N on the regression models.

This section elaborates on the experimentation and results. Each model had 31×(N+1) input features, since, N+1 days were provided as input for 31 features (Table 3). Table 5 lists the comparison of the models’ metrics with different combinations of N, K, and M for both binary classification in yellow and regression in red. The first two columns represent the values of K and M, respectively, while the remaining six columns are for the various values of N. The column for each value of N is divided into two columns for F1 and $R^{2}$, respectively. The impact of M, K, and N on F1 is shown in Figure 5, and their impact on $R^{2}$ is shown in Figure 6 using boxplots. The median value of the box plot is shown for easy comparison. A total of 432 experiments are recorded in Table 5. Here, 216 are for binary classification models with F1, and 216 are for regression models with $R^{2}$.

For **binary classification** experiments, a prediction greater than the threshold of 0.5 was considered a forest fire. It is seen in Table 5 that F1 is excellent (>0.9) for 83.4% of the experiments, good (between 0.8 and 0.9) for 13% of the experiments, and poor (between 0.5 and 0.8) for 3.6% of the experiments. This shows that forest fire prediction depends on weather features, similar to the findings in [8,9,10]. From Figure 5a, it can be seen that the median F1 is excellent (>0.9) for all values of M except for M=12 where it is 0.802. In Figure 5b, it is noticed that increasing the value of K has a negligible increase in the median value of F1. When compared with various values of N in Figure 5c, the median F1 is >0.90 and increases as the value of N increases until N=30 and then remains constant, implying that having 30 days of historical information is sufficient.

Table 5 for **regression** experiments shows that 69.4% of the experiments explain a significant amount of variance ($R^{2}$>0.75) in data, 19% of the experiments explain a good amount of variance (between 0.5 and 0.7), and 11.6% of the experiments explain little to no variance in data ($R^{2}$<0.5). When comparing the impact of M to predict the area burned in Figure 6a, it can be seen that M=12 has little to no variance explained by the models, as expected. The models could explain a significant or good amount of variance for the remaining values of M. *K* has no impact on the models’ performance, as it had negligible change as seen in Figure 6b, similar to binary classification. Figure 6c shows that as the value of *N* increases, the median $R^{2}$ gradually increases. Unlike the binary classification models, the values of N do not show an optimal upper limit for regression models.

Apart from the aforementioned experiments in Table 5, additional experiments were conducted to have a temporal split in $D_{train}$ and $D_{test}$ instead of having a random split. This ensures fire event and non-fire event data from the specified cutoff date are not present in $D_{test}$. The cutoff date was set to $January1^{st},2016$, i.e., $D_{train}$ has fire event and non-fire event data from 1998 to 2015 (17 years), while $D_{test}$ has fire event and non-fire event data between 2016 and 2018 (2 years). There was no noticeable change in the performance metrics ($R^{2}$ and F1).

## 6. Discussion

The STAS framework was found effective in the study of forest fire prediction. If data are collected from sensors other than the ones in the weather station, then the sets *W* and *S* can be replaced with the respective data. This was demonstrated in [9], where weather station data were replaced by lightning and hydrometric data. When the data are gathered from a single remote-sensing source, such as a satellite, then Algorithm 1 (K-Nearest Sensor Data Aggregation) is not needed, and the data can instead be passed to Algorithm (Event Points Extraction and Non-Event Point Extraction). There is no restriction on the number of features a data point can have in the STAS framework. Hence, univariate data can also be used.

The units for N and M depend on the forest fire disaster. The units of N should indicate the time for a forest fire to happen and M should be one unit higher. For example, the time frame in which a forest burns is given in days. Hence, units for N are in days, while M is in months. M and N are the main parameters of STAS. Multiple models trained on the STAS dataset for varying values of M can be ensembled to make predictions or single models can be used to make predictions based on the temporal change in data points.

This research agrees with the findings of previous research, which specify the use of weather data to predict forest fires [37,38,39,40,41,42,43]. It goes further by providing a framework that is repeatable with less computation time for a larger area (i.e., entire Canada) over large time frames and in real time. In addition, it presents STAS as a faster multivariate sampling technique for evolving data in the domain of natural forest fire disasters. The transformation of data by STAS to ***identify the change in features over a time frame for an event to occur*** changes the framing of the problem.

Since, non-events are sampled in the same location as events, but in a past time frame, it makes the dataset **spatially agnostic**. The only variation in the fire and non-fire data points will be the temporal change in data points. When models are trained on such a dataset, they will learn the variations in features distinguishing between events separated by a temporal difference of M. From [9], it is known that STAS does not make predictions based on seasonal differences. When STAS is provided a data source with no predictive power, then the model trained on such a dataset performs poorly.

### 6.1. STAS Parameters

Sensor data are associated with the physical location of the sensors. K specifies how many spatially distant sensors from an event affect the predictions. It was found to have a negligible impact on both the classification and severity prediction of forest fires. This is likely since the occurrence of forest fires is dependent on local conditions rather than on neighboring conditions. For this reason, one should use data available as close to a natural disaster as possible.

Natural disasters, such as forest fires, do not occur overnight. They gradually build up over time based on climatic and geological factors. Therefore, the parameter N specifies the number of historical days of information needed in a single data point to predict a natural fire disaster. Having a larger N incorporated in the model provides better results. After a certain value of N, it will no longer be viable. STAS framework can be used to determine this threshold. Since the regression model predicts severity, it does not have an upper bound for prediction. This requires more historical data to make accurate predictions.

M specifies the time difference to consider for change in features. In seasonal data, a recurring trend occurs, and for this reason, for some values of M, the models will easily distinguish between the data. In the case of forest fires, where the data also have associated seasonality, the models will easily distinguish between data for M=3,4,5,6,7,8,9,10, as there is sufficient difference in values over that time frame. Metrics for M=1,2,11,12 can be used to test if the dataset is a good predictor of natural phenomena. This is because there are little or no variations in event and non-event data. Especially, for M=12, where fire and non-fire events are from the same month, leading to similar weather patterns. This technique was applied in [9], where hydrometric data were found to be of no use, while lightning was found to be the best predictor of a forest fire. Similarly, using this technique in [10], it was found that weather data could be used for both predicting the occurrence and severity of a forest fire, while lightning data only work for predicting the occurrence. In this research, STAS produced good results for M=12 in the case of predicting the occurrence of forest fires. The value of N can be increased to yield high performance in models for M=12, as it will need more historical information.

### 6.2. Sampling

In the study of natural forest fire disasters, random sampling is not suitable, as different researchers will obtain different datasets based on the random seed. This may cause challenges when other researchers attempt to replicate the work. Further, a random instance may provide a dataset with good results while the other may not, as was seen in [12,13]. One drawback of the NearMiss approach is that the under-sampled data points in the majority class will change if new points are added or removed from either the minority class or the majority class. The algorithm needs to be re-run for the entire dataset. This may not be effective when data keep growing, as in the study of natural disasters, causing challenges of re-computation and repeatable work. In SMOTE, similar challenges to NearMiss arise. Further, the artificially added samples will add bias to the models.

STAS is suitable for under-sampling multivariate time series evolving data for natural fire disasters. In STAS, re-computation is only required for new data, unlike traditional algorithms. This reduces the computation time and resources. Furthermore, the data have a 1:1 ratio for event and non-event data, thereby producing a balanced dataset.

### 6.3. Metrics and Validation

**Figure 7:**
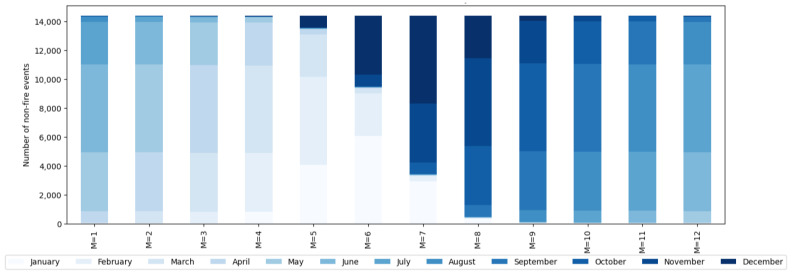
Number of non-fire events ($\overset{ˇ}{e}$) from each month for given values of parameter M.

F1 is considered an unbiased estimator for randomly sampled data. The data are not randomly sampled in STAS, but due to the change in the framing of the problem to identify the change in features over a time frame for an event to occur, it can be considered an unbiased estimator. Figure 7 shows how many non-fire events ($\overset{ˇ}{e}$) are taken from any given month for a given value of parameter M. The legend for the month is given at the bottom. It can be seen that as the value of M is varied, different months dominate in the sample for $\overset{ˇ}{e}$. Hence, it is highly encouraged to use a Mixture of Experts (MoE) approach with the same value of K and N but with M ranging from 1 to 12, one for each month. This leads the models to be trained on segments of temporally scattered data.

Possible threats to validity include selecting different model parameters and improper data preprocessing. The hidden layers, learning rates, and epochs play a critical role in model training. It is recommended that a model be trained with the specified parameters. The acquisition of data different from those specified in this paper may also potentially affect validating the results presented.

### 6.4. Limitations

This research demonstrated the applicability and procedure of STAS for multivariate seasonal time series data. It was also demonstrated in [9] that if features completely independent of forest fires are provided, i.e., hydrometric instead of lightning or weather, the models did not have good predictive power. One potential limitation of STAS is that if it is given a dataset *D* of *p* features, it would not identify which of the *p* features are important predictors. It will only be able to demonstrate if the *D* has predictive power in the *p* features for event $\hat{e}$. For identifying important features, traditional feature importance techniques can be used first for a given *D*, and then the important features can be passed as a smaller dataset to STAS.

## 7. Conclusions

In this research, the STAS framework was successfully applied to weather datasets with 31 features. This paper elaborated on the initial work presented in [8] by providing a thorough description and evaluation of the STAS framework while describing its applicability to natural disasters, such as forest fires. The K-Nearest Sensor Data Aggregation and Spatio-Temporal Agnostic Sampling algorithms were further modified in this paper for better readability and understandability. A mathematical description was provided for the extraction of event and non-event data for natural forest fire disasters. Further, a time complexity analysis with NearMiss and SMOTE showed that the STAS framework is faster.

The trained models are expected to learn the variation in features over M months and act as classifiers and severity predictors over the time frame. Further, STAS was tested on a total of 432 deep binary classification and regression experiments. It is four times greater than the experiments conducted in [8]. The additional experiments align with the findings from [8]. This research also found that N=30 is the ideal value for binary classification with CWEEDS weather sensor data.

Additionally, in this research, experimentation was also conducted where $D_{train}$ and $D_{test}$ were split over temporal values instead of being randomly split, as shown in Algorithm 4. The data between 1998 and 2015 were used to generate $D_{train}$, while the data between 2016 and 2018 were used to generate $D_{test}$. No change in performance was noted for experiments with the temporal split.

As part of future work, it is proposed that an ablation study be conducted on the features of the CWEEDS weather sensor dataset. In this research, for binary classification experimentation, a threshold of 0.5 was used to classify between fire and non-fire events. It is proposed to identify an ideal threshold for classification in future research. Further, it is proposed that models be trained on the Environment and Climate Change Canada weather station data. This allows for integration with the Environment and Climate Change Canada system to make real-time predictions. In addition, it is proposed that knowledge distillation be performed on the models to reduce their size. Finally, it is proposed to build an MoE architecture by using 12 models (one for each month) for varying values of M for a constant value of N and K.

## Figures and Tables

**Table 1:** CNFDB Dataset (*F*) Fields Used with Description.

Feature	Description
REP_DATE	Date associated with the start of forest fire ($f_{s}$)
OUT_DATE	Reported date of fire extinguished
CALC_HA	Fire size in hectares with higher precision
CAUSE	Specifies the cause of fire
GEOMETRY	2-dimensional final burnt polygonal region ($f_{g}$)

**Table 2:** CWEEDS Weather Station Metadata (*S*) Fields Used with Description.

Metadata	Description
Climate ID	Weather station ID
Location	Weather station’s latitude and longitude ($s_{l}$)
First Year	Year weather station started recording data
Last Year	Year weather station stopped recording data

**Table 3:** CWEEDS Weather Station Data (*W*) Features Used with Units.

S.No.	Feature	Preprocessing	Final Units
1	Extraterrestrial irradiance	-NA-	$\frac{kJ}{m^{2}}$
2	Global irradiance	-NA-	$\frac{kJ}{m^{2}}$
3	Direct irradiance	-NA-	$\frac{kJ}{m^{2}}$
4	Diffuse irradiance	-NA-	$\frac{kJ}{m^{2}}$
5	Global illuminance	100 lux → lux	lux
6	Direct illuminance	100 lux → lux	lux
7	Diffuse illuminance	100 lux → lux	lux
8	Zenith luminance	100 $\frac{Cd}{m^{2}}$ → $\frac{Cd}{m^{2}}$	$\frac{Cd}{m^{2}}$
9	Minutes of sunshine	-NA-	min
10	Ceiling height	10 m → m	m
11		Four digit	Digit code
12	Sky layers	sky condition	Digit code
13		codes →	Digit code
14		Digit codes	Digit code
15	Visibility	100 m → km	km
16	Thunderstorm (Weather)		Digit code
17	Rain (Weather)		Digit code
18	Drizzle (Weather)	Eight digit	Digit code
19	Snow 1 (Weather)	weather code	Digit code
20	Snow 2 (Weather)	→	Digit code
21	Ice (Weather)	Digit codes	Digit code
22	Visibility 1 (Weather)		Digit code
23	Visibility 2 (Weather)		Digit code
24	Station pressure	10 Pa → Pa	Pa
25	Dry bulb temperature	0.1 °C → °C	°C
26	Dew point temperature	0.1 °C → °C	°C
27	Wind direction	-NA-	degree
28	Wind speed	0.1 $\frac{m}{s}\to \frac{m}{s}$	$\frac{m}{s}$
29	Total sky cover	-NA-	Oktas
30	Opaque sky cover	-NA-	Oktas
31	Snow cover	-NA-	Boolean

**Table 4:** Methodology Notation/Variable Description.

Variable	Description
N	Set of natural numbers
R	Set of real numbers
∅	Empty set or empty list
N	Number of past days
M	Number of past months
K	Number of nearest sensors
*F*	Set of forest fire incidents
*f*	A single fire event
$f_{s}$	Start date of a forest fire
$f_{g}$	2-dimensional polygonal geometry of a forest fire
$f_{l}$	Center location of a polygonal region of a forest fire
*s*	A single sensor (weather station)
$s_{l}$	Location of a single sensor (weather station)
*S*	Set of all sensors (weather stations)
$S_{k}$	Set of K selected sensors (weather stations) from *S*
*w*	A single sensor (weather) data matrix
$w_{i}$	A single sensor (weather) data matrix at index *i* in $W_{k}$
*W*	Set of sensor (weather) data matrix
$W_{k}$	A list of K sensor (weather) data matrix
${\bar{w}}_{k}$	Average of K sensor (weather) data matrix
*p*	The number of features
g(.)	Mapping from set *S* to set *W*
$\hat{E}$	A list of all events (fire events)
$\hat{e}$	A single event (fire event)
$\overset{ˇ}{E}$	A list of all non-events (non-fire events)
$\overset{ˇ}{e}$	A single non-event (non-fire event)
*D*	Intermediate dataset
$D_{train}$	Training dataset without spatio-temporal information
$D_{test}$	Testing dataset without spatio-temporal information
$t_{max}$	Latest time record in days
$t_{start}$	Start date to consider for a time series
$t_{end}$	End date to consider for a time series
*A*	A dataset
$A_{maj}$	A subset of *A* with majority class values
$A_{min}$	A subset of *A* with minority class values
$A_{1}$, $A_{2}$, $A_{3}$	Set of data
*a*	A value in set *A*
*x*	Number of times to up-sample in SMOTE
$T_{dist}$	Time complexity of distance calculation
$T_{sort}$	Time complexity of sorting
$T_{extr}$	Time complexity for event extraction
$T_{interp}$	Time complexity for interpolation

**Table 5:** Representation of Model Metrics with respect to K, N, and M.

K	M	N=7		N=14		N=21		N=30		N=60		N=90	
		** F1 **	** $R^{2}$ **	** F1 **	** $R^{2}$ **	** F1 **	** $R^{2}$ **	** F1 **	** $R^{2}$ **	** F1 **	** $R^{2}$ **	** F1 **	** $R^{2}$ **
1	1	0.821	0.406	0.874	0.546	0.925	0.646	0.986	0.828	0.855	0.535	0.965	0.879
1	2	0.903	0.663	0.922	0.689	0.928	0.728	0.948	0.672	0.944	0.745	0.962	0.812
1	3	0.957	0.832	0.964	0.853	0.974	0.877	0.981	**0.907**	0.983	**0.913**	0.986	**0.924**
1	4	0.986	**0.925**	0.988	**0.931**	**0.991**	**0.944**	**0.993**	**0.944**	**0.995**	**0.970**	**0.996**	**0.972**
1	5	**0.990**	**0.952**	**0.994**	**0.955**	**0.994**	**0.959**	**0.994**	**0.964**	**0.996**	**0.976**	**0.997**	**0.933**
1	6	**0.993**	**0.971**	**0.997**	**0.966**	**0.995**	**0.976**	**0.998**	**0.975**	**0.995**	**0.980**	**0.997**	**0.977**
1	7	**0.994**	**0.958**	**0.994**	**0.967**	**0.994**	**0.968**	**0.994**	**0.964**	**0.994**	**0.967**	**0.998**	**0.985**
1	8	**0.990**	**0.948**	0.989	**0.947**	**0.991**	**0.953**	0.987	**0.944**	**0.996**	**0.968**	**0.997**	**0.963**
1	9	0.976	0.876	0.976	0.889	0.978	**0.908**	0.988	**0.936**	0.988	**0.930**	0.989	**0.941**
1	10	0.905	0.692	0.920	0.729	0.934	0.731	0.937	0.683	0.947	0.784	0.959	0.837
1	11	0.817	0.455	0.868	0.558	0.923	0.664	0.977	0.824	0.864	0.853	0.971	0.875
1	12	0.716	0.159	0.754	0.186	0.766	0.227	0.787	0.264	0.798	0.349	0.843	0.385
3	1	0.821	0.436	0.880	0.547	0.923	0.688	0.984	0.768	0.869	0.549	0.974	0.900
3	2	0.912	0.694	0.934	0.715	0.941	0.737	0.946	0.681	0.953	0.777	0.962	0.819
3	3	0.952	0.838	0.969	0.865	0.977	0.888	0.985	**0.902**	0.987	**0.905**	0.986	**0.936**
3	4	0.987	**0.923**	0.987	**0.943**	**0.994**	**0.951**	**0.995**	**0.944**	**0.994**	**0.972**	**0.998**	**0.974**
3	5	**0.991**	**0.952**	**0.993**	**0.951**	**0.992**	**0.955**	**0.995**	**0.960**	**0.995**	**0.965**	**0.995**	**0.982**
3	6	**0.994**	**0.967**	**0.996**	**0.969**	**0.997**	**0.976**	**0.996**	**0.979**	**0.994**	**0.973**	**0.999**	**0.975**
3	7	**0.994**	**0.961**	**0.997**	**0.965**	**0.995**	**0.967**	**0.995**	**0.967**	**0.997**	**0.969**	**0.999**	**0.979**
3	8	**0.990**	**0.937**	**0.994**	**0.953**	**0.994**	**0.953**	**0.997**	**0.945**	**0.997**	**0.963**	**0.997**	**0.965**
3	9	0.975	0.883	0.979	**0.900**	0.985	**0.951**	0.989	**0.931**	0.987	**0.935**	0.987	**0.940**
3	10	0.915	0.709	0.933	0.746	0.940	0.736	0.944	0.721	0.950	0.786	0.958	0.836
3	11	0.831	0.472	0.888	0.581	0.929	0.700	0.981	0.791	0.880	0.569	0.973	0.906
3	12	0.723	0.188	0.789	0.253	0.805	0.289	0.803	0.347	0.832	0.412	0.829	0.452
5	1	0.824	0.451	0.890	0.590	0.929	0.703	0.988	0.701	0.890	0.589	0.971	0.922
5	2	0.907	0.659	0.945	0.738	0.943	0.735	0.949	0.655	0.956	0.780	0.961	0.837
5	3	0.962	0.841	0.975	0.856	0.978	**0.900**	0.986	**0.919**	0.984	**0.913**	0.988	**0.928**
5	4	**0.992**	**0.931**	**0.992**	**0.951**	**0.992**	**0.957**	**0.996**	**0.947**	**0.996**	**0.969**	**0.997**	**0.981**
5	5	**0.991**	**0.951**	**0.993**	**0.963**	**0.995**	**0.963**	**0.993**	**0.964**	**0.997**	**0.969**	**0.997**	**0.976**
5	6	**0.995**	**0.977**	**0.996**	**0.972**	**0.997**	**0.974**	**0.998**	**0.958**	**0.997**	**0.974**	**0.998**	**0.980**
5	7	**0.992**	**0.967**	**0.995**	**0.965**	**0.996**	**0.971**	**0.997**	**0.973**	**0.997**	**0.973**	**0.998**	**0.982**
5	8	**0.991**	**0.902**	**0.994**	**0.950**	**0.996**	**0.953**	**0.997**	**0.953**	**0.995**	**0.976**	**0.997**	**0.968**
5	9	0.970	0.899	0.983	**0.914**	0.986	**0.951**	**0.991**	**0.931**	0.987	**0.925**	**0.992**	**0.945**
5	10	0.941	0.705	0.945	0.753	0.943	0.768	0.948	0.699	0.949	0.785	0.955	0.844
5	11	0.833	0.467	0.899	0.582	0.939	0.708	0.981	0.813	0.886	0.603	0.975	0.895
5	12	0.760	0.191	0.802	0.255	0.808	0.323	0.813	0.342	0.839	0.468	0.836	0.497

Binary classification model metrics in columns F1 and regression model metrics in columns $R^{2}$.

## Data Availability

The data presented in this study were derived from the following resources available in the public domain: CNFDB https://cwfis.cfs.nrcan.gc.ca/datamart/download/nfdbpoly [47] accessed on 5 May 2023 and CWEEDS https://collaboration.cmc.ec.gc.ca/cmc/climate/Engineer_Climate/CWEEDS_FMCEG/ [48] accessed on 5 May 2023 provided by Environment and Climate Change Canada and Natural Resources Canada, respectively. The authors confirm that the processed data supporting the findings of this study are available from the corresponding author upon reasonable request.

## Abbreviations

The following abbreviations are used in this manuscript:

BCE	Binary Cross-Entropy
BUI	BuildUp Index
CNFDB	Canadian National Fire Database
CWEEDS	Canadian Weather Energy and Engineering Datasets
CWFIS	Canadian Wildland Fire Information System
DC	Drought Code
DMC	Duff Moisture Code
FFMC	Fine Fuel Moisture Code
FWI	Fire Weather Index
ISI	Initial Spread Index
MoE	Mixture of Experts
MSE	Mean Square Error
NFDB	National Fire Data Base
RF	Random Forest
SGD	Stochastic Gradient Descent
SMOTE	Synthetic Minority Over-sampling TEchnique
STAS	Spatio-Temporal Agnostic Sampling
SVM	Support Vector Machine
